# Detection of Changes in CEA and ProGRP Levels in BALF of Patients with Peripheral Lung Cancer and the Relationship with CT Signs

**DOI:** 10.1155/2023/1421709

**Published:** 2023-02-18

**Authors:** Jingshan Huang, Kaiming Ren

**Affiliations:** Department of Thoracic Surgery, Shengjing Hospital of China Medical University, Shenyang, Liaoning Province 110004, China

## Abstract

**Objective:**

To investigate the relationship between the detection of changes in the levels of carcinoembryonic antigen (CEA) and progastrin-releasing peptide (ProGRP) in bronchoalveolar lavage fluid (BALF) and CT signs in patients with peripheral lung cancer.

**Methods:**

Retrospective analysis of 108 patients with perihilar lung cancer who attended our hospital from January 2019 to January 2022, 54 cases were randomly selected as the observation group and 50 cases as the control group. Patients in both groups received CT examination and BALF test at the same time to observe and compare the differences in serum levels, the relationship between CT signs and serum indices, and the diagnostic value of peripheral lung cancer between the two groups.

**Results:**

The serum levels of ProGrp, CEA, CA211, and NSE in the observation group were significantly higher than those in the control group, and the difference was statistically significant (*P* < 0.05). The morphology, density, mass enhancement pattern, bronchial morphology, obstructive signs, and lymph node fusion of CT signs were compared between the observation group and the control group, indicating that CT signs were more helpful for the localization, diagnosis, and staging of lung cancer. The results of ROC curve analysis showed that the AUC value of low-dose CT combined with serum ProGrp, CEA, CA211, and NSE was 0.892, sensitivity was 96.21%, and specificity of 90.05%, which were significantly higher than those of the single tests, respectively. The positive likelihood ratio was 84.41% and the negative likelihood ratio was 87.11%.

**Conclusion:**

The combination of CT signs and serum tumour markers helps to improve the detection rate, sensitivity, and specificity of lung cancer, which has a high diagnostic rate for lung cancer and may provide evidence for the early diagnosis of lung cancer.

## 1. Introduction

Peripheral lung cancer is difficult to diagnose because of the late onset of clinical symptoms and the small size of the tumour in the early stage, which can easily be overlooked by clinicians and patients, thus delaying the best time for treatment and affecting the therapeutic effect [1]. Therefore, it is important to choose a safe, effective, and convenient test to improve the diagnosis of peripheral lung cancer in order to improve the prognosis of patients. Currently, tumour markers are the main serological and biochemical indicators for clinical understanding of tumour histogenesis, cell differentiation, and cell function, and their levels can provide important references for clinical diagnosis and classification of tumours, and thus guide the formulation of treatment plans and prognosis [2]. In addition, CT has the characteristics of short acquisition time, high resolution, and better overall observation of lung cancer than other examinations, which can clearly show the morphology of cancer foci, density differences, and their relationship with blood vessels, bronchi, and other tissues [3]. At the same time, enhanced CT can further reflect the blood supply of the cancer foci to help clinical judgment of benign and malignant lesions based on the blood supply of the foci [4]. Clinical studies have concluded that tumour markers are closely related to the occurrence and development of lung cancer, most of the CT manifestations of peripheral lung cancer have nodules or masses in the peripheral lung, but they are complex, variable, overlapping, and have low specificity, so the clinical diagnosis of peripheral lung cancer often requires the combination of imaging, serum tumour markers, and the ultimate diagnosis is pathological section staining [5]. Although there are a large number of tumour markers, there are fewer specific tumour markers that can be used to reflect the condition of lung cancer, and the relevant studies are not comprehensive. Serum progastrin releasing peptide (ProGrp) and carcinoembryonic antigen (CEA) have certain clinical value for the diagnosis of lung cancer. The value of combined serum ProGrp and CEA tests in the early diagnosis of lung cancer has rarely been reported.

## 2. Materials and Methods

### 2.1. Study Subjects

Fifty-four patients with peripheral lung cancer treated in our hospital from January 2019 to January 2022 were selected as the observation group, and 50 patients with benign lung disease were selected as the healthy comparison group. All patients in our study met the diagnostic criteria for lung cancer in the Chinese Medical Association Clinical Guidelines for the Treatment of Lung *Cancer* (2018 edition) [6] and were diagnosed with lung cancer by X-ray, magnetic resonance imaging, pathological examination, and clinical confirmation; patients underwent bronchoalveolar lavage before testing, and the distal end of the fibrodectomy was wedged in the subsegment where the lesion was located, or placed in the bronchus of the segment to which it belonged if the subsegment was completely obstructed. The biopsy hole is filled with 50 ml of room temperature sterile saline for flushing and 13.3 kp of negative pressure suction, with a recovery of approximately 40%. The specimens were processed immediately after collection. After filtering through a double layer of sterile gauze, 5 ml was taken and centrifuged at 2500 pm for 10 minutes, and 1.5 ml of supernatant was stored in a refrigerator at 4°C. The precipitate was collected by centrifugation in 1.5 ml freezing tubes and stored in a -70 °C refrigerator. In the observation group, 42 males and 12 females, aged 45–83 years, were classified according to pathological tissue type. 16 small-cell lung carcinomas, 15 squamous carcinomas, and 19 adenocarcinomas. In the comparison group, there were 31 males and 19 females, aged 42–81 years.

### 2.2. Inclusion and Exclusion Criteria

Inclusion criteria: (i) all lung cancer patients had not undergone chemotherapy and radiotherapy, all patients and their families gave informed consent and signed the patient informed consent form, which was approved by the ethics committee of the hospital; (ii) eastern cooperative oncology group (ECOG) score [7]: 0–2, expected survival time ≥3 months, CT scan of head, chest, and upper abdomen, scan mode was plain + enhanced, serum tumour markers, no treatment prior to CT and serum tumour markers, and no history of other malignancies; and (iii) two or more of the tumour markers pro-GRP and CEA are simultaneously greater than the normal reference range.

Exclusion criteria: (i) patients with severe cardiac, hepatic, or renal insufficiency and patients with thyroid disorders or immune disorders; (ii) patients with severe infectious diseases, patients with coagulation disorders, and patients with pulmonary vascular disease or pulmonary encystment; and (iii) patients with severe diabetes, hypertension, cardiac diseases, and other underlying diseases requiring hospitalisation.

## 3. Methods

### 3.1. CT Examination Method

The equipment was a Siemens Definition AS+ 128-layer spiral CT machine. The patient was asked to hold his breath after calm breathing for a conventional CT scan, and then a double-phase enhancement scan was performed after contrast was injected through the elbow vein. All images were reconstructed and postprocessed to observe the morphological features of the lesion in multiple directions. The imaging features of the lesions included bronchial cut-off, density (homogeneous and heterogeneous with a clear central necrotic area), enhancement (homogeneous enhancement and heterogeneous enhancement), obstructive changes, lymph node metastases, large vessel invasion, pericardial effusion, pleural metastases, and distant metastases (including brain metastases, adrenal metastases, and bone metastases). The above results were analysed by two senior physicians in a double-blind method on the images, and consensus was reached after discussion in case of disagreement between the two.

### 3.2. Detection Method

The patient undergoes bronchoalveolar lavage prior to biopsy brushing and the distal end of the fibrinoscope is wedged in the subsegment where the lesion is located or if the subsegment is completely obstructed, placed in the bronchus of the segment to which it belongs. The biopsy hole is filled with 50 ml of room temperature sterile saline for flushing and 3.3 to 13.3 kp of negative pressure suction, with a recovery of approximately 40–60%. The specimen is processed immediately after collection. After filtering through a double layer of sterile gauze, 5 ml was taken and centrifuged at 2500 pm for 10 minutes. 1.5 ml of the supernatant was stored in a refrigerator at 4°C for centralised testing of serum tumour markers, such as CEA, NSE, and proGRP. Reference values of serum tumour markers: CEA: 0–5 ug/1; CYFRA21-1: 0–3.3 ng/m1; NSE: 0–15.2 ng/1; and proGRP: 0–35 ng/1. Any one or more of these markers outside the normal range is considered positive.

### 3.3. Observed Indicators

Positive CT scan findings were based on the following criteria: (1) 1 or more nodules ≥5 mm in diameter in the lung parenchyma; (2) solid nodules in the bronchi; a threshold of 46.0 ng/L for ProGrp, 10 ng/mL for CEA, and 15.2 ng/mL for NSE. CT combined with serum ProGrp, CEA, and NSE is considered positive if any of the tests are positive; otherwise, CT combined with serum ProGrp, CEA, and NSE is considered negative, where a positive result is predictive of lung cancer. The “gold standard” for lung cancer diagnosis: lung cancer diagnosis confirmed by pathology and imaging.

### 3.4. Statistical Analysis

All statistical data in this study were entered into an excel software by the first author and the corresponding author, and the statistical processing software was SPSS25.0 for calculation. Repeated measure analysis of variance between groups was used to measure the measurement expressed as mean ± standard deviation (*X* ± SD). Count data expressed as percentage (%) were tested by *χ2*. Univariate and logistic multivariate regression analysis was used to compare the influencing factors, and the risk factors with significant differences were screened. Correlation test using logistic regression linear correlation analysis. Included data that did not conform to a normal distribution were described by M(QR), using the Mann–Whitney test. All statistical tests were two-sided probability tests, the statistical significance was *P* < 0.05.

## 4. Results

### 4.1. Comparison of Serum Levels

**Figure 1:**
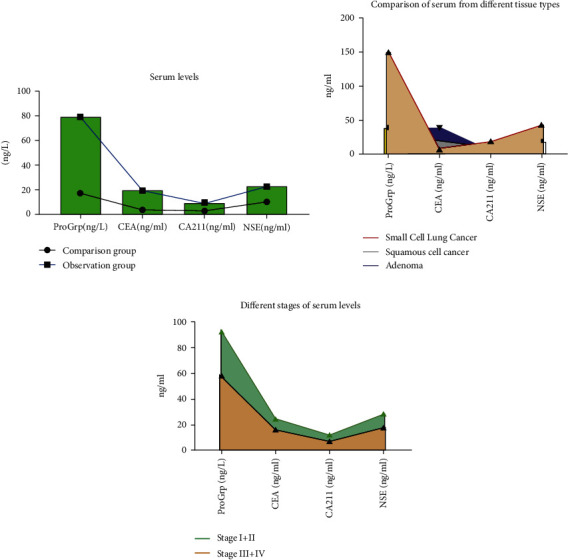
Comparison of serum levels (all serum levels data in this study were entered into excel software by the first author and the corresponding author, and the independent sample *t* test was performed using the mean ± standard deviation. The serum levels of ProGrp, CEA, CA211, and NSE were significantly higher in the observation group than in the comparison group (a) The serum levels of ProGrp, CA211, and NSE were significantly higher in the small-cell lung cancer observation group than in the squamous cancer and adenocarcinoma groups (b) and the serum levels were significantly lower in lung cancer patients in stage I and II than in patients in stage III and IV (c) with statistically significant differences (*P* > 0.05).

The serum levels of ProGrp, CEA, CA211, and NSE of patients in the observation group were significantly higher than those in the comparison group. The serum levels of ProGrp, CA211, and NSE of patients in the small-cell lung cancer observation group were significantly higher than those in the squamous carcinoma and adenocarcinoma groups, and the serum levels of patients with stage I and II lung cancer were significantly lower than those of patients with stage III and IV, with statistically significant differences (*P* > 0.05), see Figure 1.

### 4.2. Comparison of CT Signs

**Figure 2:**
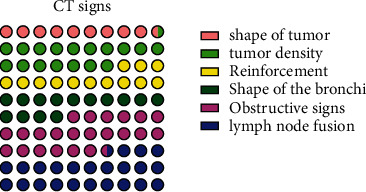
Comparison of CT signs (all CT sign data in this study were entered into excel software by the first author and the corresponding author, using integer representation and independent samples *t* test. The results showed that the morphology of the tumour, the density of the tumour, the enhancement pattern of the tumour, the morphology of the bronchi, the obstructive signs of the tumour, and the lymph node fusion of the tumour were statistically significant when compared within the observation group (*P* < 0.05).

Through the comparison of CT signs, it was found that the tumour shape, tumour density, tumour enhancement mode, bronchial shape, tumour obstructive signs, and tumour lymph node fusion were compared within the observation group, and the differences were statistically significant (*P* < 0.05), see Figure 2.

### 4.3. Comparison of CT Signs with Serum Indices

**Figure 3:**
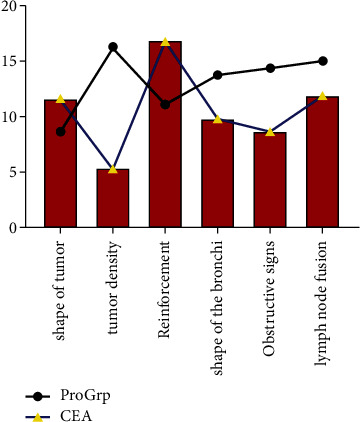
The relationship between CT signs and serum indexes (in this study, all data comparing CT signs with serum indices were entered into excel software by the first author and the corresponding author, using integer representation and independent samples *t* test. The results showed that ProGrp differed significantly in the density of the tumour and the mode of enhancement, and CEA differed statistically between lymph node swelling and no lymph node swelling (*P* < 0.05).

ProGrp showed a statistically significant difference in tumour density and enhancement pattern, while CEA showed a statistically significant difference between lymph node swelling and no lymph node swelling (*P* < 0.05) and was highly expressed in the presence of lymph node swelling, see Figure 3.

### 4.4. Diagnostic Value

The results showed that the AUC value of 0.892, sensitivity 96.21%, and specificity 90.05% of low-dose CT combined with serum ProGrp and CEA were significantly higher than those of the individual tests, and their positive likelihood ratio and negative likelihood ratio were 84.41% and 87.11%, respectively, see Table 1.

## 5. Discussion

Lung cancer is a common malignant tumour in China, with high incidence and mortality rates and is a serious threat to public health problem that threatens human health [8]. At present, surgery is an important treatment for early peripheral lung cancer, but because peripheral lung cancer has insidious onset and early symptoms are not obvious, most patients have developed to advanced stage when diagnosed, and early peripheral lung cancer is small in size and unclear in margins, which makes clinical diagnosis difficult, easily causing missed diagnosis and misdiagnosis, delaying the best treatment time and depriving patients of the possibility of surgical cure [9]. Therefore, for peripheral type lung cancer, it is not only necessary to raise patients' awareness of timely consultation, but also to select effective tools to improve the diagnostic accuracy and prognosis of patients. The radiation dose of low-dose CT is only 1/6 of that of conventional CT, but its sensitivity is comparable to that of conventional CT. It can detect overlapping, tiny nodules, reflect the nature of nodules, and is more sensitive to isolated nodules in the lung, but its specificity is relatively poor [10]. In recent years, clinical studies have concluded that tumour markers are closely related to the occurrence and development of lung cancer and have important clinical value for the early diagnosis and prognostic assessment of lung cancer, but the collection of the literature has revealed that there are relatively few reports related to lung cancer tumour markers [11].

In this study, the serum ProGrp, CEA, CA211, and NSE levels in the observation group were significantly higher than those in the healthy comparison group, indicating that the combined detection of CT signs and serum tumour markers helped to improve the detection rate of lung cancer. The specific reasons are as follows: ProGrp is a gastrointestinal hormone commonly found in various neuroendocrine-derived tumours, and clinical studies have found that it has high sensitivity and specificity for the diagnosis of small-cell lung cancer and can be used as a marker for small-cell lung cancer, which helps in the diagnosis and clinical treatment of small-cell lung cancer disease [12].CEA is an acidic glycoprotein in intestinal cancer, mainly in the embryonic stage by the small intestine and the liver. It is a broad-spectrum tumour marker and its level increases dramatically after the development of many tumours [13].CA211 is mainly found in the cytoplasm of tumour cells and can be released into the blood when tumour cells are necrotic or lysed, which is useful for the diagnosis and treatment of squamous carcinoma [14].NSE is an acidic protease that is found in neural tissue and the neuroendocrine system and mainly acts in the glycolytic pathway [15]. It was found that small-cell lung cancer can secrete NSE and release it into the blood, resulting in elevated levels, so monitoring SE is useful in the diagnosis and treatment of small-cell lung cancer, but the presence of NSE in red blood cells can lead to falsely elevated results [16].

In this study, the comparison of CT signs revealed that the morphology, density, enhancement pattern, morphology of bronchi, obstructive signs, and lymph node fusion of the mass were compared between the observation group and the comparison group, indicating that CT signs are more helpful for the localization, diagnosis, and staging of lung cancer. Currently, CT is widely used for the diagnosis of chest lesions and its diagnostic value for lung cancer is mainly its ability to clearly display the morphological features of tumours and visualize the location, size, morphology, margins, and density of tumours [17]. Reconstruction and postprocessing can also show the relationship between the tumour and surrounding tissues, as well as observe metastases in other regions, such as tumour lymph node metastases in the scanned area [18, 19]. Small-cell lung cancer originates from neuroendocrine cells and is a hypodifferentiated tumour. The tumour cells grow outside the lumen and rarely inside the lumen, forming an oblong or irregular mass in line with the long axis of the bronchus. The lumen may be narrowed by compression, with smooth mucosa and lumen patency [20, 21]. It can also cause truncation and obstructive changes in the bronchi, but tends to appear later. We found statistical differences in the morphology, density, bronchial morphology, and obstructive features of hilar lesions between the squamous and small-cell lung cancer groups, with squamous carcinomas often forming round or round-like masses in the hilum, causing bronchial truncation and significant obstructive pneumonia or obstructive atelectasis, or both [22, 23]. In our study, there was no statistically significant difference in metastasis between the squamous carcinoma group and the small-cell lung cancer group, probably due to the late clinical stage of the cases in this study and no difference in metastasis between the two.

CEA was statistically significant and highly expressed in the presence of lymph node swelling. The combination of imaging with molecular biology and molecular pathology has now enabled the understanding of the molecular pathological basis of morphological features in imaging and has played an important role in improving the diagnosis and understanding of the disease [24]. Serum tumour markers are chemical components produced by tumour cells during growth, which can be released directly into the cells or post-cellular tissues, and its abnormalities are often earlier than the symptoms, signs, and imaging manifestations of the tumour, which can be used for early detection, but local diagnosis is more difficult [25, 26]. By combining the two, the strengths and weaknesses of each can be complemented to solve the problem of localization and characterization of lung cancer and improve the correctness of the diagnosis [27].

In this study, ProGRP is a tumour marker associated with tissues of neuroendocrine origin. It has been shown that elevated serum ProGRP levels occur in a variety of neuroendocrine-derived tumours and that ProGRP is useful in the differential diagnosis of small-cell carcinoma and non-small-cell carcinoma in lung masses [28]. ProGRP has been reported to be the most sensitive marker capable of distinguishing lung cancer from benign lung disease [29]. ProGRP is elevated in the early stages of lung cancer, but because of the low incidence of lung cancer in the general population, the ProGRP program is not recommended for use in screening tests in the general population [30]. CEA is a tumour marker that is relatively sensitive to many tumours and has a role in the occurrence, efficacy observation, and prognosis of many tumours, but its specificity is not strong, its sensitivity is not high, and its role in the early diagnosis of tumours is not obvious [31]. In addition to primary colon cancer, the positivity rate of pancreatic, gastric, lung, and breast cancers is also high, generally at 80%. Because CEA is a broad-spectrum tumour marker, it is often tested in combination with other tumour markers [32].

Tumour markers are substances that are produced and secreted by cancer cells during tumour development and progression or released into the blood in response to tumour stimulation of the host, indicating the presence and growth of tumours [33]. Although serum tumour markers have some value in cancer diagnosis, pathological staging, and prognostic assessment, it is widely believed that they are susceptible to multiple factors, which may result in false positives or false negatives and affect the clinical judgment, and even though multiple serum tumour marker levels are combined, they are not at the ideal clinical level [33]. Although serum tumour markers have some value in cancer diagnosis, pathological staging, and prognostic assessment, there are various serum tumour markers that can be used for lung cancer screening, the most commonly used ones include CEA and NSE [34]. NSE is an enzyme marker that is specific for central nervous cells and endocrine tissues and has high serum levels in neuroendocrine diseases (e.g., small-cell carcinoma of the lung) and adenocarcinoma [35].

In conclusion, the combination of CT signs and serum tumour markers can help to improve the detection rate, sensitivity, and specificity of lung cancer, with a high diagnostic rate of lung cancer and can provide strong evidence for the early diagnosis of lung cancer.

## Figures and Tables

**Table 1:** Comparison of diagnostic effectiveness.

	AUC (95% CI)	Sensitivity (%)	Specificity (%)	Positive likelihood ratio	Negative likelihood ratio
CT	0.647 (0.6080.732)	55.87	77.71	78.16	82.47
CEA	0.739 (0.6870.859)	62.79	80.23	85.59	64.07
NSE	0.784 (0.7030.844)	78.16	81.25	75.36	80.23
ProGrp	0.892 (0.7750.953)	96.21	90.05	84.41	79.25
Combined detection	0.584 (0.5150.674)	49.64	74.56	70.81	87.11

## Data Availability

The experimental data used to support the findings of this study are available from the corresponding author upon request.

## References

[1] Yu A., Li Q., He J., Zhan Y. (2020). Effect of 5-line signs in the prediction of staging, progression, and prognosis of peripheral lung carcinoma: preliminary observation report. *Journal of Computer Assisted Tomography*.

[2] Shen C., Wang X., Che G. (2016). A rare case of primary peripheral epithelial myoepithelial carcinoma of lung: case report and literature review. *Medicine (baltimore)*.

[3] Pedersen J. H., Saghir Z., Wille M. M. W., Thomsen L. H., Skov B. G., Ashraf H. (2016). Ground-glass opacity lung nodules in the era of lung cancer ct screening: radiology, pathology, and clinical management. *Oncology (williston Park)*.

[4] Wang R. H., Xu K., Li L., Wu Z. F., In E. (2020). [lung cancer combined with connective tissue disease-related interstitial lung disease: ct features]. *Zhonghua Zhong Liu Za Zhi*.

[5] Tamponi M., Crivelli P., Montella R. (2021). Exploring the variability of radiomic features of lung cancer lesions on unenhanced and contrast-enhanced chest ct imaging. *Physica Medica*.

[6] Zhonghua Zhong Liu Za Zhi (2018). Chinese medical association, Chinese medical association oncology branch, Chinese medical association journal press. Chinese medical association lung cancer clinical diagnosis and treatment guidelines (2018 edition). *Chinese Journal of Oncology*.

[7] Boran E., Ramantani G., Krayenbühl N. (2019). High-density ecog improves the detection of high frequency oscillations that predict seizure outcome. *Clinical Neurophysiology*.

[8] Koike Y., Aokage K., Ikeda K. (2020). Machine learning-based histological classification that predicts recurrence of peripheral lung squamous cell carcinoma. *Lung Cancer*.

[9] Omori T., Aokage K., Nakamura H. (2019). Growth patterns of small peripheral squamous cell carcinoma of the lung and their impacts on pathological and biological characteristics of tumor cells. *Journal of Cancer Research and Clinical Oncology*.

[10] Cmi Q., Sperandeo M. (2021). Letter to the editor regarding the article: “vascularization of primary, peripheral lung carcinoma in ceus - a retrospective study (n = 89 patients)” by findeisen h et al. *Ultraschall Med*.

[11] Li Q., Sang S. (2020). Diagnostic value and clinical significance of combined detection of serum markers cyfra21-1, scc ag, nse, cea and progrp in non-small cell lung carcinoma. *Clin Lab*.

[12] Li J., Chen Y., Wang X., Wang C., Xiao M. (2021). The value of combined detection of cea, cyfra21-1, scc-ag, and pro-grp in the differential diagnosis of lung cancer. *Translational Cancer Research*.

[13] Li Y., Li M., Zhang Y. (2021). Age-stratified and gender-specific reference intervals of six tumor markers panel of lung cancer: a geographic-based multicenter study in China. *Journal of Clinical Laboratory Analysis*.

[14] Holdenrieder S., Von pawel J., Dankelmann E. (2008). Nucleosomes, ProGRP, NSE, CYFRA 21-1, and CEA in monitoring first-line chemotherapy of small cell lung cancer. *Clinical Cancer Research*.

[15] Ishibashi N., Maebayashi T., Aizawa T., Sakaguchi M., Okada M. (2019). Serum tumor marker levels at the development of intracranial metastasis in patients with lung or breast cancer. *Journal of Thoracic Disease*.

[16] Mehta A., Parkash A., Bhatia M. (2021). Cross-sectional study to establish the utility of serum tumor markers in the diagnosis of lung cancer. *Asian Pac J Cancer Prev*.

[17] Kadoya N., Tanaka S., Kajikawa T. (2020). Homology-based radiomic features for prediction of the prognosis of lung cancer based on ct-based radiomics. *Med Phys*.

[18] Xu T., Zhang X., Zhang S. (2020). Imaging features and prognostic value of 18f-fdg pet/ct detection of soft-tissue metastasis from lung cancer: a retrospective study. *Bmc Cancer*.

[19] Koo C. W. (2021). Editorial comment: lobectomy versus sublobar resection-how preoperative ct features can determine how much to resect in stage ia non-small cell lung cancer. *American Journal of Roentgenology*.

[20] Wang T., Gong J., Duan H. H., Wang L. J., Ye X. D., Nie S. D. (2019). Correlation between ct based radiomics features and gene expression data in non-small cell lung cancer. *Journal of X-Ray Science and Technology*.

[21] Opoka L. M., Szturmowicz M., Oniszh K. (2019). Ct imaging features of thin-walled cavitary squamous cell lung cancer. *Advances in Respiratory Medicine*.

[22] Sun Z., Hu S., Ge Y. (2020). Radiomics study for predicting the expression of pd-l1 in non-small cell lung cancer based on ct images and clinicopathologic features. *Journal of X-Ray Science and Technology*.

[23] Bianconi F., Palumbo I., Fravolini M. L. (2019). Texture analysis on [18f]fdg pet/ct in non-small-cell lung cancer: correlations between pet features, ct features, and histological types. *Mol Imaging Biol*.

[24] Peng Y., Wang Y., Hao X., Li J., Liu Y., Wang H. (2017). [utility of multiple increased lung cancer tumor markers in treatment of patients with advanced lung adenocarcinoma]. *Zhongguo Fei Ai Za Zhi*.

[25] Chen Z., Liu X., Shang X., Qi K., Zhang S. (2021). The diagnostic value of the combination of carcinoembryonic antigen, squamous cell carcinoma-related antigen, cyfra 21-1, neuron-specific enolase, tissue polypeptide antigen, and progastrin-releasing peptide in small cell lung cancer discrimination. *Int J Biol Markers*.

[26] Li Q., Kim J., Balagurunathan Y. (2017). Ct imaging features associated with recurrence in non-small cell lung cancer patients after stereotactic body radiotherapy. *Radiat Oncol*.

[27] Kamran S. C., Coroller T., Milani N. (2020). The impact of quantitative ct-based tumor volumetric features on the outcomes of patients with limited stage small cell lung cancer. *Radiat Oncol*.

[28] Wang J., Shi G., Zhang S. (2010). [clinical value of serum tps, cea, pro-grp and cyfra21-1 in patients with lung cancer]. *Zhongguo Fei Ai Za Zhi*.

[29] Wang H., Zhang J., Li X. (2020). The utilization pattern of serum tumor markers in lung cancer patients: a population-based retrospective descriptive study. *Journal of Clinical Laboratory Analysis*.

[30] De Kock R., Borne B. V. D., Soud M. Y. E. (2021). Circulating biomarkers for monitoring therapy response and detection of disease progression in lung cancer patients. *Cancer Treatment and Research Communications*.

[31] Jia H., Zhang L., Wang B. (2019). The value of combination analysis of tumor biomarkers for early differentiating diagnosis of lung cancer and pulmonary tuberculosis. *Ann Clin Lab Sci*.

[32] Wang L., Wang D., Zheng G. (2016). Clinical evaluation and therapeutic monitoring value of serum tumor markers in lung cancer. *Int J Biol Markers*.

[33] Zeng Q., Liu M., Zhou N., Liu L., Song X. (2016). Serum human epididymis protein 4 (he4) may be a better tumor marker in early lung cancer. *Clinica Chimica Acta*.

[34] Liang X., Zhu J., Cai M. (2020). Progrp as a novel biomarker for the differential diagnosis of medullary thyroid carcinoma in patients with thyroid nodules. *Endocrine Practice*.

[35] Okamura S., Fujiwara Y, Nagata K. (2019). Multiple osteolytic bone and lung metastases from prostate cancer including small cell carcinoma with marked increases in cea and pro-grp. *Urology Case Reports*.

